# Compounds of Essential Oils from Different Parts of *Cinnamomum cassia* and the Perception Mechanism of Their Characteristic Flavors

**DOI:** 10.3390/foods14203570

**Published:** 2025-10-20

**Authors:** Yuhua Huang, Wei Wang, Xuan Xin, Shanghua Yang, Weidong Bai, Wenhong Zhao, Wenbin Ren, Mengmeng Zhang, Lisha Hao

**Affiliations:** 1 College of Light Industry and Food Technology, Zhongkai University of Agriculture and Engineering, Dongsha Street 24, Guangzhou 510225, China; 15119309783@163.com (Y.H.);; 2 College of Chemistry and Chemical Engineering, Zhongkai University of Agriculture and Enineering, Dongsha Street 24, Guangzhou 510225, China; 3 School of Food Science and Technology, Jiangnan University, Wuxi 214122, China

**Keywords:** cinnamon essential oil, flavor profile, olfactory receptors, GC-MS, molecular dynamics simulation, electronic nose

## Abstract

This study investigated the differences in key volatile organic compounds (VOCs) and flavor characteristics between essential oils (CEOs) from cinnamon bark and leaf. The volatile compounds of essential oils extracted from *Cinnamomum cassia* (Xijiang) bark (CEOP) and leaf (CEOY) by hydrodistillation were identified using GC-MS. The results showed that the extraction rates of CEOP and CEOY were 1.56% ± 0.02 and 0.83% ± 0.01 (*n* = 3), respectively. CEOP and CEOY consisted of 45 and 50 compounds, respectively. Odor activity value (OAV) analysis indicated that cinnamaldehyde (OAV = 935), α-caryophyllene (OAV = 77), and borneol (OAV = 4) played key roles in shaping the aroma of CEOP. Meanwhile, cinnamaldehyde (OAV = 849), nerolidol (OAV = 107), and α-caryophyllene (OAV = 58) were the major contributors to the flavor of CEOY. Electronic nose (E-nose) analysis revealed that sensors W5S and W1W were important for detecting aromatic compounds. Sensory evaluation showed that CEOs differed significantly in spicy, floral, and grassy aromas. These differences may be related to the concentrations of compounds such as cinnamaldehyde, α-caryophyllene, and nerolidol, as well as their interactions with olfactory receptors such as OR2W1 and OR1D2. Cinnamaldehyde activates TRPA1 and TRPV1 to elicit the perception of spiciness. Thus, CEOP may be suitable for baked goods, and CEOY may be suitable for ice cream and beverages. In conclusion, this study provides a theoretical foundation for the precise application of CEOs as condiments in food.

## 1. Introduction

Cinnamon is a spice plant and one of the most economically important plant resources, with widespread distribution in China, India, Madagascar, and Sri Lanka [1]. Cinnamon essential oil (CEO) is a natural volatile oil extracted from the bark and leaves, containing aldehydes, terpenes, and aromatic compounds. CEO exhibits various pharmacological properties, including antibacterial, antioxidant, anti-inflammatory, anti-anxiety, anti-tumor effects and regulation of glucose and lipid metabolism, attracting increasing attention [2,3,4,5]. As a condiment, CEO is also commonly used in cola-type beverages, coffee, candy, food preparation, and in the cosmetics and fragrance industries [4,6,7]. Cinnamon bark, which constitutes the secondary phloem of the tree trunk, contains well-developed oil cells and resin. Oil cells are more densely distributed in the phloem than in the parenchyma tissue of leaf [8]. Therefore, cinnamon bark contains more oil than leaf but regenerates more slowly. Unlike leaves, cinnamon bark can be used directly as a spice [9]. Due to cost considerations, extracting essential oil from cinnamon leaves has become routine in many areas. However, essential oils derived from different plant parts differ significantly in composition and flavor characteristics. Previous studies have shown that cinnamon bark oil and leaf oil from Sri Lankan types, such as *C. verum* and *C. sinharajaense*, differ significantly in sensory flavor characteristics [1]. These differences directly affect the taste profile and application scenarios of the essential oils. Therefore, understanding the variations in flavor compound composition and sensory characteristics between cinnamon bark and leaf essential oils is crucial for the precise application of CEO in high-value utilization. Although many studies have investigated the compositions of volatile compounds in cinnamon bark oil and leaf oil separately, differences in species, origin, extraction methods, and other factors lead to considerable variations in the chemical composition of CEOs [10,11]. Consequently, data from different studies are often difficult to compare directly. As a result, relying solely on existing studies makes it challenging to comprehensively understand the variations in compound composition and flavor characteristics of essential oils extracted from cinnamon bark and leaf.

When we smell, aromatic compounds enter the oral and nasal passages and reach the olfactory epithelium in the upper nasal cavity. They then bind to olfactory receptors (ORs) on receptor cells, triggering various types of olfactory neurons to generate electrical signals and transmit information to the brain, enabling the recognition and analysis of scents [12]. Humans possess approximately 390 ORs, which can be classified into broad-spectrum and narrow-spectrum types based on the range of odor molecules they detect [13]. Broad-spectrum receptors (OR1A1, OR1D2, OR1G1, and OR2W1) can respond to a variety of structurally distinct odor molecules, forming complex combinatorial codes, while specific receptors (OR5M3 and OR5AC2) primarily recognize a small set of specific or closely related molecules [14]. The main flavors of CEO are spicy, sweet, and floral [6,15]. Transient receptor potential A1 (TRPA1) and transient receptor potential vanilloid 1 (TRPV1) have been reported to be associated with the spiciness of CEO. OR2W1 can recognize “spicy, cinnamon, clove” notes, while OR1A1, OR1A2, and OR1D2 respond to floral and sweet aromas [16,17,18,19]. In recent years, the mechanisms underlying interactions between ORs and aromatic compounds have attracted considerable attention. A thorough understanding of these interactions is essential for predicting and controlling the release or retention of aroma molecules by receptors [20]. However, there is currently no clear research on how the key flavor components of CEO interact with these receptors. Therefore, exploring the interaction patterns between the distinctive flavor molecules of CEOs and olfactory receptors could provide insight into the physiological basis of their unique aromas [21].

Although many different types of cinnamon exist worldwide, *C. cassia* and *C. verum* are the most widely cultivated. According to statistics, China produces nearly 80% of the world’s CEO, with *C. cassia* being the dominant species, mainly distributed in Guangdong and Guangxi provinces. However, few studies have examined the differences in compound composition and flavor characteristics between *C. cassia* bark and leaf oils. One of the most widely cultivated *C. cassia* varieties in these areas is “Xijiang Cinnamon” [22,23]. Therefore, exploring the variations in flavor constituents between Xijiang cinnamon bark and leaf essential oils is representative. In this study, CEOs were prepared from Xijiang cinnamon bark and leaves, and GC-MS was employed to analyze their volatile compounds, revealing differences in volatile organic compounds (VOCs). Multi-technology integration, including electronic nose analysis and sensory evaluation, was also applied. Furthermore, by investigating the interactions between the key VOCs in CEOs and ORs, we identified the physiological basis underlying the flavor differences of CEOs. This study elucidates the material basis and characteristic aroma formation mechanisms of CEOs, providing insights into the molecular perception mechanisms that shape their distinctive spicy aroma, while offering strategies for regulating flavor intensity and designing targeted flavor-enhancing products.

## 2. Materials and Methods

### 2.1. Materials and Chemicals

Xijiang cinnamon bark and leaves were harvested in June 2023 from Qianguan Town, Yunan District, Yunfu, Guangdong Province, China (22°52′ N, 111°31′ E). The region has a subtropical monsoon climate characterized by an average annual temperature of 21 °C and annual precipitation of 1400 mm. The soil in the cinnamon garden is red loam. All cinnamon plants were cultivated under identical agricultural practices, bark and leaf samples were harvested according to a standardized picking. After harvesting, the cinnamon bark and leaf were stored indoors in a 20–25 °C environment with a relative humidity of 50–60% and used within three months.

A mixture of n-alkanes (C8-C20, analytical grade) was supplied by Sigma-Aldrich (St. Louis, MO, USA). Ethanol was obtained from Tianjin Yongda Chemical Reagent Co., Ltd. (Tianjin, China). 2-Octanol (chromatographic grade), used as an internal standard, was purchased from Shanghai Macklin Biochemical Technology Co., Ltd. (Shanghai, China).

### 2.2. Cinnamon Essential Oil Extraction

Cinnamon bark and leaf were ground and then passed through a 60-mesh sieve. A 500 g sample was placed in a 10 L vapor pot (Chengdu Chengxiang Technology Co., Ltd., Chengdu, China) with 4 L of water and soaked for 12 h. The condenser reflux tube was installed and always maintained an inflow of condensate water at 20–25 °C. The electric stove was initially set to 2200 W. When distillation began and pure condensate started dripping into the receiving tube, the power was reduced to 500 W, and distillation continued for 5 h. After distillation, the condensate was collected and centrifuged at 1000× *g* for 5 min at 25 °C. The yellow oily fraction was collected in a 125 mL pear-shaped separatory funnel and left to stand for 12 h, and then the yellow oil liquid was released from the bottom of the funnel, stopping the collection when a white layered interface was observed. This was collected in a brown bottle and stored at 10–12 °C with a relative humidity of 40–50% and used within three months (Figure S1). The extraction yield of essential oils was calculated using Equation (1).

M(%) = [m_2_/m_1_] × 100% (1)

where M(%) is the extraction yield of the oil, m_2_ is the weight of extracted oil (g), and m_1_ is the initial weight of raw material (g).

### 2.3. Identification of Volatile Compounds by Gas Chromatography-Mass Spectrometry (GC-MS)

With minor modifications, the previously reported method was used for GC-MS analysis [11]. The analysis was conducted on an Agilent 7890B gas chromatography system equipped with an HP–5MS elastic quartz capillary column (30 m × 0.25 mm × 0.25 μm, 19091S–433, J&W Scientific, Folsom, CA, USA) and an Agilent 5977A mass spectrometry detector. The CEO samples were mixed with the internal standard (2-octanol) and filtered through a 0.22 μm organic membrane before injection. The GC-MS conditions were as follows: the initial oven temperature was set at 40 °C for 2 min, then increased to 80 °C at 3.0 °C/min, further increased to 120 °C at 4.0 °C/min, then to 180 °C at 6.0 °C/min, and finally to 230 °C at 10.0 °C/min, which was maintained for 30 min. The injection volume was 1.0 μL, the inlet temperature was set at 250 °C, and a split ratio of 3:1 was used. High-purity helium (>99.999%) served as the carrier gas at a constant flow rate of 4 mL/min. The mass spectrometry settings were as follows: ion source temperature, 230 °C; quadrupole temperature, 150 °C; scan range 20–400 m/z in full-scan mode; and ionization voltage, 70 eV. Each CEO sample was analyzed in triplicate. The detected compounds were compared with the National Institute of Standards and Technology (NIST) spectral library, and only compounds with a similarity of >85% were selected. Normal alkanes (C_10_–C_28_) were used to calculate the retention index (RI), which was then compared with the standard RI values https://webbook.nist.gov/ (accessed on 1 May 2025). The RI was calculated using Equation (2) [24]:

RI = 100n + 100 [(T_a_ − T_n_)/(T_n+1_ − Tn)] (2)

where RI is the retention index, T_n_ is the retention time of normal alkanes C_n_ with n carbon atoms, T_n+1_ is the retention time of normal alkanes C_(n+1)_ with n + 1 carbon atoms, and T_a_ is the retention time of the compound in the sample between T_n_ and T_n+1_.

#### 2.3.1. Qualitative and Quantitative Analysis and Odor Activity Value Calculation

Semi-quantitative analysis was performed using the internal standard method, with cyclohexanone as the internal standard. Quantification was based on the ratio of the peak area of each detected compound in the total ion chromatogram to that of the internal standard. The content of each compound was calculated using Equation (3):

Ci(μg/g) = (S_1_/S_2_) × (C_1_/C_2_) (3)

where Ci is the mass concentration of the compound to be tested (μg/g), C_1_ is the concentration of 2-octanol, C_2_ is the concentration of the sample, S_1_ is the peak area of the internal standard (2-octanol), and S_2_ is the peak area of the compound to be tested.

#### 2.3.2. Odor Activity Value Calculation

The odor activity value (OAV) was used to evaluate the contribution of individual volatile compounds to the overall aroma. The OAV of each compound was calculated using Equation (4):

OAV = N/T(4)

where N is the concentration of the volatile compound (μg/g), and T is the odor threshold of that compound. The T values were obtained from the literature.

### 2.4. E-Nose Analysis

The electronic nose system consists of 10 sensors (PEN3, Airsense Analytics Co., Ltd., Schiwerin, Germany). First, 5 mL samples of CEOP and CEOY were placed in two headspace vials and incubated in a water bath at 30 °C for 30 min, after which the headspace was collected for analysis. The electronic nose probe was then inserted to draw the headspace air for testing. Measurement conditions were as follows: sampling interval of 1 s per measurement, sensor self-cleaning time of 60 s, sensor zeroing time of 5 s, sample preparation time of 5 s, and injection flow rate of 400 mL/min. The total experimental analysis time was 60 s, and the data from the steady curve between 57 and 59 s were used for analysis. Each sample was measured in triplicate. Sensors were cleaned and normalized before and after each measurement.

### 2.5. Sensory Evaluation

The sensory evaluation panel consisted of 15 researchers (10 women and 5 men, aged 22–27), all of whom had prior experience with sensory analysis of aromatic food ingredients. Before the evaluation, all participants signed informed consent forms, were fully briefed on the procedures, and received systematic training. Appropriate protocols were implemented to protect participants’ rights and privacy throughout the study. All panelists completed a two-week training program, which included aroma reference compound familiarization, terminology alignment, intensity scaling practice using representative essential oil samples, and consensus development on aroma descriptors. The characteristic aromas of CEO included “herbal”, “woody”, “pungent”, “smoky”, “floral”, “spicy”, and “sweet”. A 0.1 g of CEOP and CEOY was placed in brown glass vials, sealed, and equilibrated at 25 °C for 1 h before testing. Panelists were asked to sniff each sample and score the intensity of the specified aromatic characteristics. Scores of 0–2 indicated no or very weak aroma, 2–5 indicated moderate intensity, and 5–10 indicated strong to extremely strong aroma. To avoid panelist interaction, sensory evaluations were carried out in separate booths in a controlled setting (25 ± 1 °C and 35–50% relative humidity). All evaluations were conducted in a uniform lighting and ventilation environment to eliminate contextual bias, and the order in which the samples were presented was randomized to minimize order effects. To minimize olfactory fatigue, panelists were instructed to rest for at least 30 s between samples and sniff coffee powder. Each evaluation was conducted in triplicate.

### 2.6. Molecular Docking

Molecular docking was performed following the method described by Zhao et al. [25]. SDF files for human olfactory receptors and sensory receptors were obtained from the protein database https://www.uniprot.org/ (accessed on 1 June 2025) and https://www.rcsb.org/ (accessed on 1 June 2025). The following receptors were identified: OR1A1 (UniProt ID: Q9P1Q5), OR1A2 (UniPort ID: Q9Y585), OR2W1 (UniPort ID: Q9Y3N9), OR1D2 (UniPort ID: P34982), OR5M3 (UniPort ID: Q8NGP4), OR5AC2 (UniPort ID: Q9NZP5), TRPA1 (UniProt ID: O75762), and TRPV1 (UniProt ID: Q8NER1). The structure of the human olfactory receptor protein was downloaded in PDB format from AlphaFold (version v2.2.0). The source organism was homo sapiens. The calculation pretest data were taken from the AlphaFold protein structure database. The 3D structures of small molecules were downloaded from PubChem (https://pubchem.ncbi.nlm.nih.gov/) (accessed on 27 June 2025). Then, Molecular docking was performed using AutoDock 1.5.7 and AutoDock Vina (The Scripps Research Institute, La Jolla, CA, USA) on small molecules and protein crystals after dehydration and hydrogenation pretreatment. The binding energy of each docking configuration was recorded. The pdbqt file was converted to a pdb file and then loaded into PyMOL (version 2.4, Schrödinger, LLC., New York, NY, USA.) to display the binding patterns. Discovery Studio (version 2024, BIOVIA, Omaha, NE, USA) was used to review amino acid residues in the receptor active region.

### 2.7. Molecular Dynamics (MD) Simulations

Molecular dynamics simulations were carried out using Gromacs [26]. The force field parameters were generated using the Gromacs pdb2gmx tool and the AutoFF website. The molecular properties of the receptor protein were assigned with the CHARMM36 force field, while those of the ligand were described using the CGenff force field. The system was solvated in a 1 nm cubic TIP3P water box [27]. To verify electrical neutrality, counterions were added with the gmx genion tool; seven Cl^−^ were added to counterbalance the positive charges within the system. Long-range electrostatic interactions were treated with the particle mesh ewald (PME) method, with a cutoff distance of 1 nm. Bond constraints were applied using the SHAKE algorithm, and the Verlet leapfrog algorithm was employed with an integration step size of 1 fs. Before the molecular dynamics simulation, the system underwent energy optimization, consisting of 3000 steps of steepest descent followed by 2000 steps of conjugate gradient optimization. Equilibration (200 ps) was carried out using modified Berendsen thermostat coupling at 300 K and isotropic Berendsen pressure coupling at 1 atm. The optimization procedure first constrained the solute while minimizing water molecules, then constrained the counterions for minimization, and finally minimized the entire system without constraints. The simulation was conducted under NPT conditions at 310 K and constant pressure for 100 ns. Trajectory analyses were carried out using built-in Gromacs tools: g-RMSD for root mean square deviation (RMSD), g-RMSF for root mean square fluctuation (RMSF), g-HBonds for hydrogen bonds (HBonds), g-Rg for radiuses of gyration (Rg), and g-SASA for solvent accessible surface area (SASA).

### 2.8. Statistical Analysis

Data are presented as means ± standard deviation (SD) of three replicates from independent experiments. One-way analysis of variance (ANOVA) followed by multiple comparisons was performed using SPSS (version 21.0, Inc., Chicago, IL, USA). to determine significant differences among groups. The results were considered statistically significant when *p* < 0.05. All graphical visualizations were generated with Origin (version 2024, Northampton, MA, USA) and Graphpad Prism 9.5 (GraphPad Software, LLC, San Diego, CA, USA). The multivariate analyses, including principal component analysis (PCA) and orthogonal partial least-squares discrimination analysis (OPLS-DA), were performed using SIMCA (version 14.1, Umet-rics, Umeå, Sweden). Molecular dynamics simulations were carried out with GROMACS (version 2022, Developer Teams from KTH Royal Institute of Technology & Uppsala University, Stockholm, Sweden & Uppsala, Sweden).

## 3. Results

### 3.1. Extraction and Component Analysis of Essential Oil

#### 3.1.1. Essential Oil Yield of Different Parts

**Figure 1:**
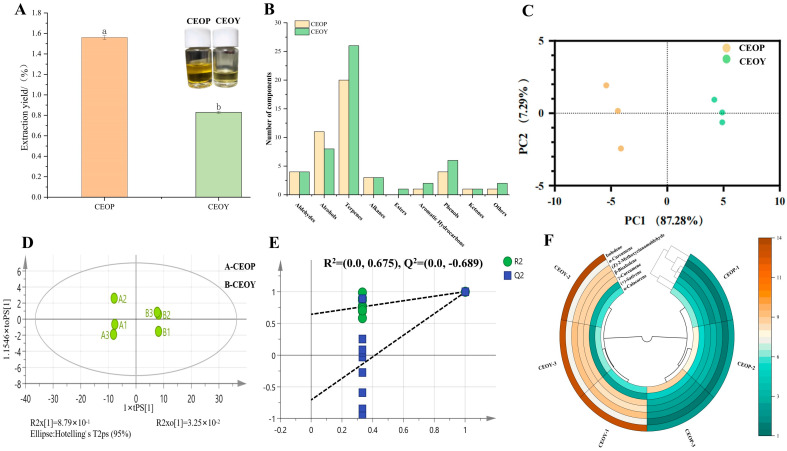
(**A**) Extraction yield of the essential oils from different parts (*p* < 0.05); (**B**) Types and number of volatile components of cinnamon essential oils from different parts; (**C**) PCA plot of two cinnamon essential oils; (**D**) OPLS-DA plot of cinnamon essential oils from different parts; (**E**) 200 cross permutation tests analysis diagram; (**F**) Heatmap of differential aroma components between essential oils of different parts of cinnamon (OVA ≥ 1, *p* < 0.05).

Hydrodistillation is the most commonly used method for extracting essential oils and has been widely applied in industrial production. Therefore, the CEO was extracted by hydrodistillation. As shown in Figure 1A, the extraction yields of CEOP and CEOY were 1.56% and 0.83%, respectively. Furthermore, CEOP exhibited a deep yellow color, whereas CEOY appeared pale yellow, indicating clear distinctions between the oils. Xijiang cinnamon bark includes abundant oil cells and resin, with oil cell density slightly higher in the phloem than in the leaf parenchyma tissue [14]. Similarly, the extraction yield of oil from *C. cassia* bark and leaves was 1.65% and 0.24%, respectively [10]. More advanced extraction methods, such as supercritical fluid extraction and ultrasonic-assisted extraction, have been reported to achieve essential oil extraction yields ranging from 2% to 7%, which are higher than those obtained in our study [11,28]. However, the equipment costs and operational complexity of these processes are considerably higher than those of hydrodistillation. For economic reasons, these advanced techniques have not yet been widely adopted in practical production. Therefore, the CEOP and CEOY obtained in this study are more representative of essential oils used in practical applications.

#### 3.1.2. Identification of Volatile Compounds in CEOs

GC-MS was applied to identify the volatile compounds in CEOs. According to the analysis (Table 1), a total of 64 compounds were detected across the samples. Aldehydes were the predominant class in all CEOs, followed by terpenes, phenols, and alcohols (Figure 1B). CEOP consisted of 45 compounds, including 4 aldehydes, 11 alcohols, 20 terpenes, 3 alkanes, 1 aromatic hydrocarbon, 4 phenols, 1 ketone, and 1 other compound. CEOY consisted of 50 compounds, including 4 aldehydes, 8 alcohols, 26 terpenes, 3 alkanes, 1 ester, 2 aromatic hydrocarbons, 4 phenols, 1 ketone, and 1 other compound. In comparison, *C. cassia* bark oil obtained by hydrodistillation consisted of 15 compounds, including 4 aldehydes, 4 oxygenated monoterpenes, 2 sesquiterpene hydrocarbons, and 5 other oxygenated compounds [11]. Meanwhile, the number of compounds identified in CEO was more diverse than that reported by Wang et al. [29] for hydrodistillation, which included 9 alcohols, 6 aldehydes, 1 alkane, 1 carboxylic acid, 3 esters, 1 ether, and 1 ketone in *C. cassia* leaf oil. This difference may be attributed to differences in detection methods and the regional cultivation environment.

The major compounds of CEOP are cinnamaldehyde, (+)-d-cadinene, α-muurolene, copaene, and 1-isopropyl-4,7-dimethyl-1,2,3,5,6,8a-hexahydronaphthalene. In CEOY, the main compounds are cinnamaldehyde, copaene, 1-isopropyl-4,7-dimethyl-1,2,3,5,6,8a-hexahydronaphthalene, (+)-d-cadinene, and cyclopentane. Furthermore, benzaldehyde, cubenene, and γ-muurolene were detected in CEOP but not in CEOY, whereas camphene, zonarene, calarene, gossonorol, and (Z)-1-methyl-4-(6-methylhept-5-en-2-ylidene) cyclohex-1-ene were found in CEOY but not in CEOP. *C. cassia oil* is predominantly composed of cinnamaldehyde, which is consistent with previous studies [30].

However, other components also exhibit differences. For example, hydrodistillation of *C. cassia* bark oil yielded primarily cinnamaldehyde (78.31%), along with (Z)-2-methoxycinnamaldehyde (8.55%) and copaene (1.50%) [11]. Similarly, Jadhav et al. [31] identified (E)-cinnamaldehyde (76.98%) as the dominant component, followed by α-copaene (6.36%) and a naphthalene derivative (4.32%). However, the content of (Z)-2-methoxycinnamaldehyde was notably lower in our CEOs. In fact, oils from other cinnamon varieties besides *C. cassia* also exhibit such differences. For example, the primary constituents of oil extracted from *C. zeylanicum* leaves are trans-cinnamaldehyde (16.25%), eugenol (79.75%), and (3-Ethoxy-hexa-1,5-dienyl)-benzene (1.14%) [29]. In addition to eugenol and cinnamaldehyde, Schmid reported that oil from the same variety also included β-caryophyllene (4.1%) [32]. The diverse composition of the CEO is primarily influenced by plant variety, source location, geographical environment, and extraction method [1]. Although these compounds are present at lower concentrations than cinnamaldehyde, they may significantly affect the overall flavor of CEO. Therefore, understanding their contribution is essential for the precise application of the CEO in food processing.

According to PCA (Figure 1C), the contribution rates of PC1 and PC2 were 87.28% and 7.29%, respectively, totaling 94.57%. This indicates these two PCs can effectively reflect the overall variation in CEOs. The PCA plot showed that the samples were well separated, suggesting significant differences in flavor between CEOP and CEOY. Specifically, CEOP was located on the left half of Figure 1D, whereas CEOY appeared on the right half. OPLS-DA was also applied to identify CEOs. The model exhibited strong performance, with an independent variable fitting index (*R*^2^*_x_*) of 0.911, *R*^2^*_y_* of 0.999, and *Q*^2^ of 0.993. Values of *R*^2^ and *Q*^2^ above 0.5 indicated that the model was reliable. The VIP values for all compounds were determined using OPLS-DA (Table S1), with a VIP ≥ 1 indicating a major role in separating CEOs from different parts. Compounds with VIP ≥ 1 included 4 aldehydes, 8 alcohols, 26 terpenes, 3 alkanes, 1 ester, 2 aromatic hydrocarbons, and 2 phenols. Notably, nerolidol, 2-hydroxybenzaldehyde, τ-muurolol, and cinnamaldehyde exhibited high VIP values, showing that these compounds contribute considerably to the differentiation of CEOP and CEOY and can serve as key markers for identification. This is caused by the compounds in CEOs and the weight coefficients in the OPLS-DA.

After 200 permutation tests (Figure 1E), the intercept of *Q*^2^ on the vertical axis was −0.689, lower than the original OPLS-DA *Q*^2^, confirming the model’s validity. Furthermore, the *R*^2^ and *Q*^2^ points on the far right exceeded those on the left, and the regression line connecting *R*^2^ and *Q*^2^ intersected the horizontal axis below zero. This suggests that the *Q*^2^ value decreases under permutation, validating the model and confirming the role of the Y-variable classification in identifying the volatile aromatic components of CEOs. In addition, a heat map analysis was performed on common compounds with VIP > 1 and *p* < 0.05 (Figure 1F). Isoledene, α-curcumene, (Z)-2-methoxycinnamaldehyde, β-bisabolene, and γ-curcumene showed variations between CEOP and CEOY, highlighting their importance in distinguishing the two oils.

Table 2 shows the OAV values calculated from the flavor thresholds and concentrations of volatile compounds. CEOP contained five compounds with OAV > 1: cinnamaldehyde, α-caryophyllene, benzaldehyde, α-pinene, and borneol. CEOY contained six compounds with OAV > 1: cinnamaldehyde, α-caryophyllene, borneol, α-pinene, nerolidol, and 2-hydroxybenzaldehyde. Based on OAV values, the flavor of CEOP is primarily affected by cinnamaldehyde and α-caryophyllene, while the flavor of CEOY is mainly affected by cinnamaldehyde, α-caryophyllene, and nerolidol. Cinnamaldehyde, α-caryophyllene, α-pinene, and borneol are important contributors in both oils. These key compounds play a major role in shaping the distinct flavors of the two CEOs.

Notably, the five compounds with VIP > 1 that distinguish CEOP and CEOY all have OAV values < 1 (Table S1). The absence of threshold values for sesquiterpenes in essential oils causes OAV values below one, indicating that they make only a minor direct contribution to the overall aroma and are not the key aromatic compounds of CEO. Reported that in *C. cassia* bark oil, the highest OAV value was observed for anethole (394), followed by cinnamaldehyde (213), nonanal (149), and 1-octen-3-ol (127) [33]. Furthermore, 15 flavor compounds (OAV > 1) were identified, including eugenol, hexanal, benzaldehyde, 4-aminobenzaldehyde, and borneol. Other studies have also shown that compounds such as hexanal, nonanal, α-caryophyllene, benzaldehyde, hexanoic acid, heptanoic acid, ethyl cinnamate, trans-cinnamaldehyde, and coumarin have OAV values greater than one in *C. cassia* bark oil [30]. In contrast, our study did not detect hexanal, nonanal, hexanoic acid, ethyl cinnamate, or coumarin. This discrepancy may be due to differences in GC-MS instrumentation and detection methods. Overall, the OAV values of key flavor compounds differ extensively in CEOs, leading to flavor distinctions between the two extraction parts.

**Table 2:** OAV values of key volatile compounds in two cinnamon essential oils.

No	Compound	Odor Threshold mg/L ^c^	CEOP	CEOY
1	Cinnamaldehyde	0.75	935.12 ± 98.22	849.10 ± 45.52
2	α-Caryophyllene	0.16	77.87 ± 7.06 ^a^	58.62 ± 11.93 ^b^
3	Benzaldehyde	0.75	3.57 ± 0.97	NA
4	Borneol	0.18	4.05 ± 0.83 ^a^	13.77 ± 1.11 ^b^
5	α-Pinene	0.16	2.87 ± 2.75 ^a^	9.00 ± 5.62 ^b^
6	Nerolidol	0.1	NA	107.00 ± 8.00
7	2-Hydroxy Benzaldehyde	0.34	NA	2.67 ± 0.20

CEOP: Cinnamon bark essential oil, CEOY: Cinnamon leaf essential oil. The data are shown as means ± SD. NA, not detected. ^a–b^ Means in the same row with different superscript letters differ significantly (*p* < 0.05). ^c^ Reference on odor thresholds in water [34].

### 3.2. Electronic Nose Analysis of Aromatic Components in CEOs

**Figure 2:**
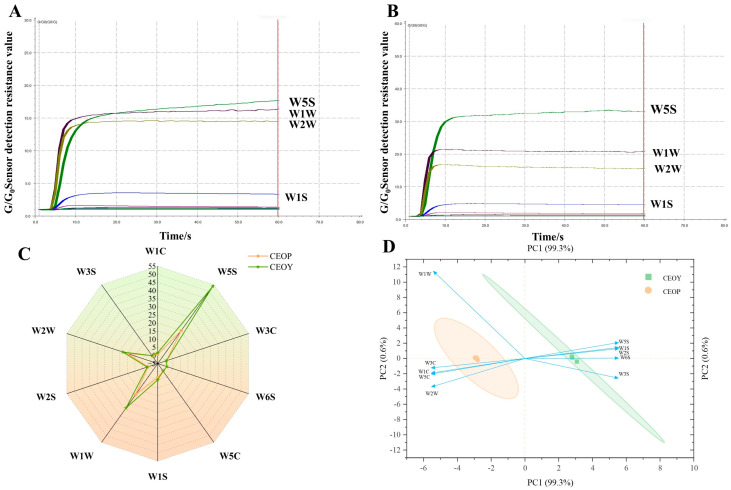
(**A**) Electronic nose test typical chromatograms of essential oil extracted by cinnamon bark essential oil, green line for W5S sensor, purple line for W1W sensor, yellow line for W2W sensor, blue W1S sensor; (**B**) Electronic nose test typical chromatograms of essential oil extracted by cinnamon leaf essential oil; (**C**) Electronic nose radar diagram of rose essential oil volatiles extracted by different parts; (**D**) Electronic nose PCA diagram of rose essential oil volatiles extracted by different parts, orange areas for CEOP confidence interval, green areas for CEOY confidence interval.

The CEOs extracted from various parts of cinnamon were analyzed using an electronic nose, and the results are shown in Figure 2A,B. The response value of CEOY was higher than that of CEOP, as determined by comparing the resistance change (G) induced by CEO passing through the electronic nose sensor with the resistance change (G_0_) caused by air. CEOP may contain a higher concentration of aldehydes, which are strongly polar and tend to undergo chemical adsorption with metal oxides on the sensor surface. This interaction forms stable bound structures, resulting in considerable resistance changes (increased G/G_0_). However, cinnamaldehyde has a relatively high molecular weight and low volatility, which may minimize sensor contact.

In contrast, CEOY contains multiple low-polarity terpenoid compounds, including α-pinene, camphene, 1,13-tetradecadiene, zonarene, and caryophyllene oxide. These compounds are characterized by low polarity and high volatility, favoring physical adsorption on sensor surfaces and causing rapid but transient resistance changes (lower G/G_0_) [35]. Radar maps are vital for assessing taste differences by displaying multidimensional data. Figure 2C shows the electronic nose radar map of CEOs from various components. CEOP had the highest response on sensor W5S, followed by W1W, W2W, W1S, W2S, W6S, and W3S (Table S2). CEOY also showed the highest response on W5S, greater than CEOP, suggesting more nitrogen oxides. W1W, W2W, W1S, W2S, and W1C followed, all with higher values than CEOP. The response values of W5C, W3C, W6S, and W3S were relatively similar.

It can be implied that CEOs extracted from various parts are most sensitive to sensors W5S, W1W, and W2W, with significant differences observed between the response of W5S and W1W. Sensor W5S is sensitive to both nitrogen oxides and aromatic compounds. CEOY contains a broader range of aldehydes, terpenoid oxides, and large-molecule conjugated terpenoids than CEOP. The methoxy group of (Z)-2-methoxycinnamaldehyde in CEOY increases the electron density of the benzene ring, enhancing its electrical interaction with the metal oxide surface of W5S [36]. Terpenoid oxides, such as caryophyllene oxide, possess epoxy or peroxy bonds with electron-accepting characteristics comparable to nitrogen oxides, thereby inducing a cross-response in W5S. Moreover, large-molecule conjugated terpenes in CEOY, including β-bisabolene, α-curcumene, and γ-curcumene, may further activate W5S [37]. As a result, CEOY exhibits higher response values than CEOP in sensors W5S and W1W. In addition, the wider range of terpene compounds in CEOY accounts for the higher response of W1W. Overall, the electronic nose distinguishes CEOs with various compositions mainly through five sensors: W5S, W1W, W2W, W1C, and W2S. The remaining five sensors also respond to compounds, but their variations are minor and do not effectively differentiate samples.

To examine the influence of different extraction locations on the flavor of CEOs, PCA analysis was performed on the electronic nose sensor response data (Figure 2D). CEOs were distinguished across different extraction locations along the horizontal axis of the scatter plot. PC1 explained 99.3%, PC2 explained 0.6%, and together the first two principal components accounted for 99.9%. The much higher contribution of PC1 compared with PC2 indicates that CEOP contains a broader accumulation of volatile components than CEOY. This suggests considerable variation in volatile compounds between the groups, as reflected by the CEO signal points without overlap. Combining the VIP and *p* values from OPLS-DA (Table S1) reveals that sensors W5S and W1W play an important role in distinguishing CEOs from different parts. These findings indicate that the electronic nose data can capture the main characteristics of volatile compounds, confirming significant differences in the aroma of CEOs. This is consistent with the GC-MS results of volatile compound analysis.

### 3.3. Sensory Evaluation Analysis

**Figure 3:**
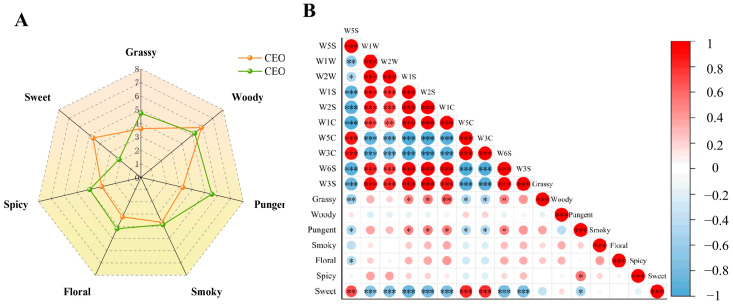
(**A**) Radar plots of the scores of two cinnamon essential oils evaluated for sensory characteristics; (**B**) Pearson correlation analysis of electronic nose and sensory evaluation of two cinnamon essential oils. * *p* ≤ 0.05, ** *p* ≤ 0.01, *** *p* ≤ 0.001.

The working mechanism of an electronic nose relies on changes in electrical impulses caused by chemical adsorption, which are principally sensitive to specific functional groups [38]. In contrast, human olfactory receptors operate through a combination coding strategy, whereby a single odorant molecule activates multiple receptors to produce distinct neural patterns, enabling the perception of subtle differences in complex odors [12]. Consequently, quantitative descriptive analysis (QDA) was applied to evaluate the sensory characteristics of CEOs and clarify their aromatic properties (Figure 3A). Spicy, sweet, and woody notes were identified as the key olfactory attributes of CEOP. This may be related to compounds such as cinnamaldehyde and a-caryophyllene, which contribute to the spicy, sweet, and woody notes. As a primary component, cinnamaldehyde imparts a warm, sweet, and rich flavor without pungency. The woody note is mainly derived from terpenoids and aromatic compounds. CEOP contained higher levels of woody aroma compounds, including copaene, α-caryophyllene, and γ-muurolene, than CEOY. Notably, α-caryophyllene also exhibited a higher OAV value in CEOP. However, CEOY displayed a wider variety of terpenoids, such as camphene, (Z)-1-methyl-4-(6-methylhept-5-en-2-ylidene)cyclohex-1-ene, caryophyllene oxide, and zonarene, which were not detected in CEOP.

The major olfactory characteristics of CEOY include floral, grassy, and pungent notes. Nerolidol has an OAV value of 107 in CEOY, and its aroma is typically floral. CEOP lacks nerolidol, which might explain the stronger floral notes in CEOY. CEOY also includes α-pinene and borneol, both contributing a fresh herbal aroma. These compounds have higher OAV values than those in CEOP and together enhance the herbal character of CEOY [39]. In addition, compounds such as 2-hydroxybenzaldehyde may account for the more pungent aroma of CEOY compared with CEOP. The smoky aroma intensity of the CEOs is relatively similar. Cinnamon bark oil and leaf oil also show significant differences in other varieties of cinnamon.

For instance, the bark oil of *C. verum* presents a cinnamon-spicy aroma, whereas its leaf oil has a pleasant geranium-like aroma. In contrast, bark and leaf oils of *C. sinharajaense* from Sri Lanka exhibit different characteristics, with camphoraceous and unpleasant leafy notes [1]. Brnawi et al. reported that Sri Lankan cinnamon bark oil has stronger sensory acceptance in dairy products, whereas leaf oil is limited in application due to off-odors [40]. Essential oils with different flavors have different applications. Based on the flavor distinctions between CEOY and CEOP, we infer that CEOP is more suitable for baked food, providing a typical warm cinnamon spice, and may also be utilized in Indian-style chili recipes [15]. In contrast, CEOY is well-suited for ice cream and desserts, offering a fresher and more unique floral note than regular cinnamon powder, and can also be applied in tea beverages to add a grassy taste.

Associations analysis is combined with human and intelligent senses (Figure 3B), reflecting both objective and subjective factors to better reveal the connection between sensory evaluation and the electronic nose for the aroma of CEOs. The 10 sensors of the electronic nose are strongly associated with spicy, grassy, pungent, and sweet aromas. Pungent and sweet aromas showed significant positive correlations with W5S, W5C, and W3C, implying that aromatics detected in CEOs, such as nitrogen oxides, olefinic aromatic components, and ammonia, contribute to this strong correlation. In contrast, sweet aroma exhibited a negative correlation with W1W, W2W, W1S, W2S, W1C, W6S, and W3S (*p* < 0.001). It is suspected that increasing amounts of inorganic sulfur compounds, organic sulfur compounds, short-chain alkanes, aromatic compounds, hydrogen compounds, long-chain alkanes, and aliphatic components in CEOs may collectively diminish their pleasant aroma. CEOY contains more types of aliphatic, aromatic, and terpenoid compounds than CEOP, which might explain why CEOP shows a sweet aroma. Short-chain alkanes, aromatic compounds, and hydrogen compounds in CEOs contributed to their pungent aroma, as did W1S, W2S, W6S (*p* < 0.05), and W1C (*p* < 0.01).

### 3.4. Molecular Docking Analysis Between the Key Aroma Compounds and ORs

#### 3.4.1. Comparison of Binding Energies Between Different ORs and Aroma Compounds

**Figure 4:**
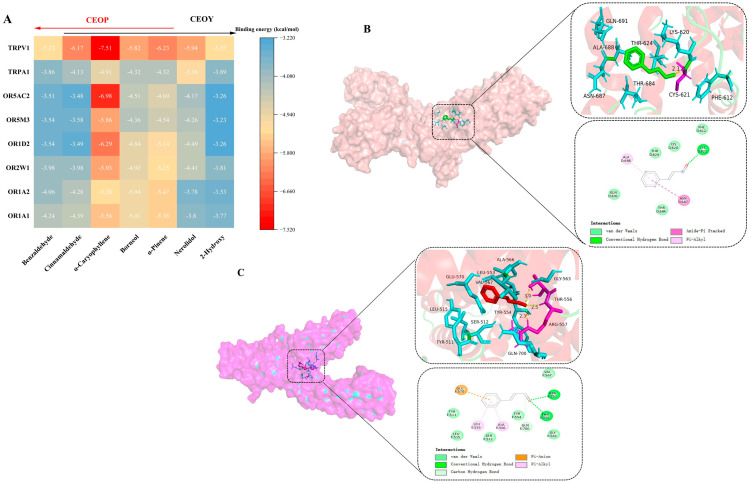
(**A**) The binding energy of key volatile compounds in cinnamon essential oil to six ORs, TRPA1 and TRPV1; (**B**) simulation results for molecular docking between cinnamaldehyde and TRPA1; (**C**) TRPV1. CEOP: cinnamon bark essential oil, CEOY: cinnamon leaves essential oil.

Molecular docking, as a computational simulation tool, can visually depict the process and outcome of aroma molecules binding to receptors [41]. The binding energy obtained from molecular docking serves as a crucial quantitative indicator for evaluating the strength of interaction between ligand molecules (flavor compounds) and biomolecules (receptor proteins). Significant differences were observed in the binding energies between various receptors and distinct volatile molecules (Figure 4A).

The binding energies ranged from −7.51 kcal/mol to −3.26 kcal/mol, indicating that the receptors and compounds can bind spontaneously. Among these compounds, those with lower binding energies tend to interact more strongly with the TRPV1 receptor, which is responsible for perceiving spicy aromas. Cinnamaldehyde exhibited low binding energy with TRPV1. TRPV1 and OR2W1 were both associated with spicy perception, and these two compounds showed higher binding energies with these receptors compared to others. This explains why CEOs exhibit a spicy aroma.

CEOP has a stronger spicy aroma than CEOY, which may be due to the higher OAV values of compounds such as α-caryophyllene, which bind more strongly to spicy receptors. Furthermore, α-caryophyllene and nerolidol show stronger binding affinities with OR1D2, which perceives floral aromas, and OR1A2, which detects sweet herbal aromas, explaining the floral and sweet aromas in CEOs. α-Caryophyllene also has a strong affinity for OR5AC2, which is associated with woody aromas, and OR1D2 for floral aromas. Its strong binding enables more effective receptor activation, leading to distinctive sweet and woody aromas. The OAV value of α-caryophyllene is higher in CEOP than in CEOY, which explains why CEOP scores higher than CEOY in sensory evaluations for sweet and woody aromas. Nerolidol, which has a high OAV value in CEOY, contributes unique floral and grassy aromas, but it is absent in CEOP, explaining the more pronounced floral and grassy aromas in CEOY. Additionally, although benzaldehyde was not detected in CEOY and 2-hydroxy benzaldehyde was absent in CEOP, their binding energies with receptors were not significantly different, and their OAV values in both CEOs were relatively low compared with other compounds.

#### 3.4.2. Types of Forces Between ORs and Aroma Compounds

Molecular docking not only allows investigation of the interactions between characteristic aroma compounds and olfactory receptors but also highlights the specific regions where these interactions occur, which is essential for understanding how volatile compounds bind to olfactory receptors. Table 3 shows the key residues involved in the binding of different aromatic compounds in CEOs to ORs.

The results show that distinct aromatic compounds interact with Val203 (OR1A1), Tyr258 (OR1A2), Phe206 (OR1A2), Gly108 (OR2W1), Leu199 (OR1D2), Phe207 (OR1D2), Leu255 (OR1D2), and Tyr259 (OR1D2). The key binding regions for olfactory receptors and ligands are distributed across transmembrane domains TM3, TM4, and TM5. The amino acids in these domains play critical roles in forming interaction forces with ligands, consistent with the findings of Xiao et al. [42]. Except for α-caryophyllene and α-pinene, most aromatic compounds can form hydrogen bonds. For instance, 2-cyanophenol forms hydrogen bonds with Gly202 and Asn109 (OR1A1); nerolidol interacts with Thr239, Ser242, and Arg122 (OR1A2) and Gly263 (OR2W1); cinnamaldehyde binds Tyr182 (OR1D2); benzaldehyde interacts with Tyr257 (OR5M3); and borneol forms hydrogen bonds with Thr111 and Ala107 (OR5AC2), among others. Although α-caryophyllene does not form hydrogen bonds with ORs, it shows strong hydrophobic interactions. As a sesquiterpene, α-caryophyllene lacks polar groups (hydroxyl, amino, or carboxyl) and thus cannot form hydrogen bonds with ORs [42].

Nevertheless, its binding energy to ORs is considerably higher than that of other compounds. This is primarily due to hydrophobic contacts, van der Waals forces, and π-π stacking. Hydrophobic amino acid residues create a nonpolar environment that stabilizes the binding of odor molecules [43]. For example, the hydrophobic region formed by Pro138, Cys141, Phe61, Ile49, and Thr57 allows 2-phenylethanol to form a stable complex with OR1A1. Similarly, although citral does not form hydrogen bonds with OR1A2, it exhibits lower binding energy than other ORs due to hydrophobic interactions with ten amino acid residues [42]. Therefore, α-caryophyllene achieves stronger binding to ORs than other compounds, primarily owing to hydrophobic interactions involving its carbon-carbon double bonds and cyclic structure [25].

Cinnamaldehyde has the highest OAV value among CEO compounds, and the spicy aroma is both the main characteristic and the most distinguishable flavor of CEOs. Therefore, we illustrate the interaction patterns of cinnamaldehyde with the spicy aroma sensory receptors TRPA1 and TRPV1 (Figure 4B,C), while the interaction forces between cinnamaldehyde and other ORs are shown in Figure S2. Cinnamaldehyde forms hydrogen bonds with Cys621 (TRPA1) and interacts with Gln691, Thr684, Asn687, Phe612, Lys620, Thr62, and Ala688, all of which affect the channel. It also forms hydrogen bonds with Thr556 and Arg557 (TRPV1) and interacts with Tyr511, Leu515, Leu553, Ser512, Ala566, Tyr554, Gln700, Gly563, Val567, and Glu570, all affecting the channel function. As an electrophilic compound, cinnamaldehyde can covalently modulate the channel by binding to cysteine residues (Cys621, Cys641, Cys665) or Lys710 in the hTRPA1 cytoplasmic NH2-terminal domain [44].

Notably, cinnamaldehyde is the only one among the ten key volatile compounds that forms hydrogen bonds with Cys621 [44]. In contrast, nerolidol exhibits strong binding affinity through two hydrogen bonds and hydrophobic interactions with ten amino acid residues, yet it does not interact with Cys621. TRPA1 and TRPV1 are activated and their ion channels open in response to compounds such as cinnamaldehyde, α-pinene, α-caryophyllene, and nerolidol, leading to the perception of spiciness in the brain [45]. In conclusion, investigating the binding energies and interaction forces between distinct aromatic compounds and ORs provides deeper insight into the mechanisms underlying the aroma of CEOs from different parts.

### 3.5. MD Simulations to Explore the Binding Mechanisms

Molecular dynamics simulations provide a detailed description of the dynamic interactions between aromatic compounds and receptors at the molecular level, revealing temporal evolution over various time scales [46]. Molecular dynamics provides a more comprehensive understanding of the binding process than the static interactions observed in energy-minimized ligand–receptor complexes during molecular docking [25]. In order to elucidate the specific interaction mechanisms of cinnamaldehyde with TRPA1 and TRPV1, molecular dynamics simulations were conducted on the complexes formed between cinnamaldehyde and these spicy sensory receptors, exploring their binding states at the molecular level.

**Figure 5:**
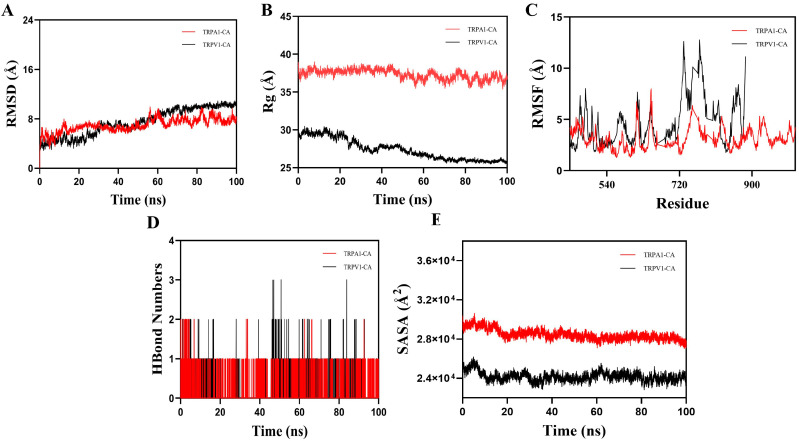
The results of MD simulations (**A**) Root mean square deviation values; (**B**) Radiuses of gyration index; (**C**) Root mean square fluctuation distribution; (**D**) The number of hydrogen bonds; (**E**) Solvent accessible surface area. CA: cinnamaldehyde.

Root means square deviation (RMSD) is a key metric for evaluating the stability of protein receptors and cinnamaldehyde conformations, as well as the deviation of atomic positions from their initial coordinates. As shown in Figure 5A, the TRPA1–cinnamaldehyde (TRPA1-CA) complex reached convergence around 15 ns, with minor fluctuations at 55 ns, and ultimately stabilized at an RMSD of approximately 8.2 Å. In comparison, the TRPV1-CA complex converges around 35 ns, with slight fluctuations at 60 ns, and stabilizes at an RMSD near 10.4 Å. These results indicate that the TRPA1-CA complex attains equilibrium more rapidly, suggesting a faster stabilization of its structure. The radius of gyration reflects the overall compactness of the protein (Figure 5B). The TRPA1-CA system exhibited stable variations after 50 ns, with an average value of 36 Å, indicating that the complex underwent minor structural adjustments at the beginning of the simulation before settling into a stable conformation. Conversely, the TRPV1-CA system showed consistent variations after 60 ns, with an average value of around 26 Å and a noticeable decreasing trend during the simulation. This suggests that the TRPA1-CA complex stabilizes faster than the TRPV1-CA complex following initial conformational changes, as cinnamaldehyde locates an optimal binding site within TRPA1. Root means square fluctuation evaluates the thermal mobility or structural flexibility of atoms. A higher RMSF value indicates greater structural adaptability, whereas a lower RMSF value suggests structural rigidity.

As shown in Figure 5C, residues 612–620 in the TRPA1-CA complex, along with the expected flexible regions at the protein terminus (743–771, 820–837, 923–946), exhibited high flexibility. In the TRPV1-CA complex, residues 612–621, 641–656, 719–822, and 850–871 were more adaptable than those in the TRPA1-CA complex. This may be due to the presence of additional hydrogen bonds or other stabilizing structural interactions in the TRPV1-CA system, leading to enhanced flexibility.

After evaluating the number of hydrogen bonds during a 100 ns simulation (Figure 5D), it was found that the TRPV1-CA system formed more hydrogen bonds than the TRPA1-CA system. This result is consistent with the molecular docking results and the changes observed in RMSF. In addition, the solvent-accessible surface area (SASA) is a useful measure of protein hydrophobicity; the higher the SASA value, the greater the molecular contact area with the aqueous solution and the more loosely organized the molecule. Analysis of the SASA showed that the TRPA1-CA system had a higher SASA value than the TRPV1-CA system (Figure 5E), indicating a more disorganized complex. According to previous reports, camphor possesses strong hydrophobic characteristics and may interact with the TRPV1 channel in the transmembrane region TM2–4 [47]. S-nitrosylation activates cysteine residues, which in turn activate TRPV1 [48]. The strong flavor and cooling sensation of camphor are partly due to its inhibitory effect on TRPA1.

In general, the TRPA1 binding site is located in the intracellular N-terminal ankyrin repeat domain (ARD) multipocket [49]. Cinnamaldehyde binds to its electrophilic sensitivity site (Cys621) through an initial conformational expansion before rapidly stabilizing, thereby exposing additional hydrophobic residues and increasing hydrophilicity [44]. The TRPV1 binding site lies within a single pocket of the voltage-sensing domain (VSD), which spans the transmembrane regions S1–S4. Upon cinnamaldehyde binding, the S4 helix contracts inward, making the pocket more compact and reducing solvent exposure [50]. The conformational expansion of TRPA1 exposes intracellular phosphorylation sites, thereby enhancing channel opening and explaining the pungent sensation induced by cinnamaldehyde. In contrast, the compact TRPV1 conformation strengthens the heat sensation by maintaining a tight lipid sheath around the transmembrane region, enabling cinnamaldehyde to synergistically enhance the perception of heat [51,52].

## 4. Conclusions

In summary, although the compositions of Xijiang cinnamon bark and leaf essential oils are comparable in terms of the compounds they contain, the relative amounts differ. Isoledene, α-curcumene, (Z)-2-methoxycinnamaldehyde, β-bisabolene, and γ-curcumene are the key components that distinguish the CEOs. In flavor, CEOP presents a spicy, woody, and sweet aroma, whereas CEOY exhibits a grassy and pungent aroma, with sweetness being the most noticeable distinction. The interaction of α-pinene, cinnamaldehyde, and nerolidol with OR1A2, OR1D2, and TRPV1 may account for these physiological distinctions. This study not only provides new directions for improving the flavor quality of CEO in various applications but also contributes to a deeper understanding of olfactory mechanisms in flavor science and how the complex aroma of CEO affects spicy perception at the molecular level.

## Figures and Tables

**Table 1:** Chemical content, aroma description in essential oils by different parts of cinnamon.

No	Compound	CAS	RI		Concentration (μg/g)		Aroma Description
			Calculated Value	Literature Value	CEOP	CEOY	
Alcohols							
1	τ-Muurolol	19912-62-0	1471	1606	9.83 ± 3.26 ^a^	4.49 ± 0.95 ^b^	Herbal, spicy, sweet
2	Caryophyllenyl alcohol	913176-41-7	1428	-	1.19 ± 1.13	3.59 ± 1.07 ^b^	-
3	(3E,7E)-1,5,5,8-Tetramethylcycloundeca-3,7-dienol	28446-26-6	1447	1618	1.79 ± 0.95	ND	-
4	Junenol	472-07-1	1457	1588	0.51 ± 0.41	ND	-
5	2,4-Quinolinediol	86-95-3	1482	-	0.45 ± 0.26	ND	-
6	Bicyclo[2.2.1]heptan-2-ol,1,7,7-trimethyl-,(1S-endo)-	464-45-9	1140	1124	0.31 ± 0.19 ^a^	5.68 ± 0.12 ^b^	Balsamic
7	Ethylene glycol	14912-44-8	1304	1322	0.60 ± 0.30 ^a^	1.66 ± 0.11 ^b^	-
8	α-Cadinol	481-34-5	1477	1610	1.45 ± 0.70 ^a^	6.08 ± 0.70 ^b^	Herbal
9	Borneol	507-70-0	1140	1136	0.73 ± 0.15 ^a^	2.48 ± 0.20 ^b^	Balsam, camphor, herbal, woody
10	Nerolidol	7212-44-4	1425	1514	ND	10.70 ± 0.80	Floral, green, waxy, citrus, woody
11	α-Bisabolol	30159-13-8	1304	-	ND	4.93 ± 1.47	-
12	(5S,6R,7S,10R)-7-Isopropyl-2,10-dimethylspiro[4.5]dec-1-en-6-ol	72203-99-7	1438	1572	3.27 ± 2.15	ND	-
13	(1S,3aS,4S,5S,7aR,8R)-5-Isopropyl-1,7a-dimethyloctahydro-1H-1,4-methanoinden-8-ol	21966-93-8	1449	-	3.26 ± 2.06	ND	-
Aldehydes							
1	Cinnamaldehyde	104-55-2	1249	1249	701.34 ± 73.67	636.83 ± 34.14	Spicy, sweet, cinnamyl, aldehydic
2	Benzenepropanal	104-53-0	1150	1160	1.21 ± 1.21	2.78 ± 1.93	Green
3	Benzaldehyde	100-52-7	-	-	0.68 ± 0.73	ND	Fruity, sweet, almond, nutty
4	(Z)-2-Methoxycinnamaldehyde	76760-43-5	1421	1463	2.45 ± 0.35 ^a^	8.70 ± 1.48 ^b^	Sweet, cinnamon, spicy, oily, woody
5	2-Hydroxy Benzaldehyde	90-02-8	-	-	ND	0.91 ± 0.07	Medicinal
Terpenes							
1	Copaene	3856-25-5	1473	1472	45.54 ± 20.82	36.94 ± 1.61	Woody
2	Cubenene	29837-12-5	1405	1512	7.32 ± 3.06	ND	Spicy
3	α-Calacorene	21391-99-1	1410	1513	6.34 ± 2.62	6.19 ± 1.17	Woody
4	α-Caryophyllene	6753-98-6	1357	1418	12.46 ± 1.13 ^a^	9.38 ± 1.91 ^b^	Sweet, woody, spicy
5	γ-Curcumene	451-55-8	1494	1487	0.93 ± 0.36	7.48 ± 0.45	-
6	β-Bisabolene	495-61-4	1391	1485	0.78 ± 0.40	8.06 ± 1.19	Balsamic, herbal
8	α-Muurolene	10208-80-7	1374	1471	55.82 ± 10.37 ^a^	26.41 ± 2.34 ^b^	Balsamic, herbal
9	γ-Muurolene	30021-74-0	1444	1444	7.63 ± 1.22	ND	Woody, herbal
10	Isoledene	95910-36-4	1280	1373	2.92 ± 0.54 ^a^	13.60 ± 0.25 ^b^	-
13	α-Pinene	7785-70-8	-	-	0.46 ± 0.44 ^a^	1.44 ± 0.90 ^b^	Herbal, spicy
14	Camphene	79-92-5	-	-	ND	4.85 ± 0.11	Woody
15	1,13-Tetradecadiene	21964-49-8	1454	1385	ND	3.48 ± 1.57	-
16	(E,Z)-α-Farnesene	1000293-03-2	1486	-	ND	1.79 ± 0.89	-
17	(Z)-1-Methyl-4-(6-methylhept-5-en-2-ylidene)cyclohex-1-ene	13062-00-5	1405	1478	ND	6.26 ± 0.49	-
18	Bicyclo[7.2.0]undec-4-ene,4,11,11-trimethyl-8-methylene-,[1R-(1R*,4Z,9S*)]-	118-65-0	1335	1383	ND	5.12 ± 0.12	Woody, spicy
19	Bicyclo[3.1.1]hept-2-ene,2,6-dimethyl-6-(4-methyl-3-pentenyl)-	17699-05-7	1347	1403	ND	5.34 ± 0.42	-
20	Caryophyllene oxide	1139-30-6	1436	1537	ND	8.32 ± 1.92	Woody, sweet spicy
21	Zonarene	41929-05-9	1386	-	ND	8.26 ± 1.26	-
22	(+)-Sativene	3650-28-0	1316	1396	4.29 ± 1.35 ^a^	2.37 ± 0.38 ^b^	-
23	(+)Cuparene	16982-00-6	1474	1488	ND	5.26 ± 0.88	-
24	β-Cadinene	523-47-7	1454	1472	3.88 ± 0.21 ^a^	8.04 ± 0.28 ^b^	Woody
25	α-Corocalene	20129-39-9	1459	1605	2.03 ± 1.09	ND	-
26	β-selinene	17066-67-0	1377	1436	0.51 ± 0.24	ND	Herbal
27	(+)-d-Cadinene	483-76-1	1400	1497	64.46 ± 18.02 ^a^	34.31 ± 0.56 ^b^	Herbal
28	α-curcumene	644-30-4	1376	1453	0.83 ± 0.19 ^a^	7.07 ± 2.19 ^b^	-
29	1H-Cyclopropa[a]naphthalene,1a,2,3,5,6,7,7a,7b-octahydro-1,1,7,7a-tetramethyl-,(1aR,7R,7aR,7bS)-	17334-55-3	1342	1407	ND	6.78 ± 0.23	-
30	1,2,4-Metheno-1H-indene	22469-52-9	1379	1377	4.80 ± 1.34 ^a^	1.11 ± 0.66 ^b^	-
Alkanes							
1	Pentane	107-83-5	-	-	3.94 ± 1.29 ^a^	6.99 ± 0.72 ^b^	-
2	Cyclohexane	499-97-8	-	-	1.07 ± 0.95	ND	-
3	Cyclopentane	96-37-7	-	-	3.10 ± 2.85 ^a^	16.79 ± 1.77 ^b^	-
4	Gossonorol	92691-77-5	1468	1625	ND	7.35 ± 0.65	-
Esters							
1	(E)-Dodec-2-en-1-yl propyl carbonate	1000372-79-9	1453	-	ND	7.41 ± 0.28	-
Aromatic Hydrocarbons							
1	O-Eugenol	579-60-2	1304	1412	0.32 ± 0.06 ^a^	2.34 ± 0.17 ^b^	Spicy
2	Phenanthrene,7-ethenyl-1,2	1686-67-5	1626	1884	ND	1.16 ± 0.20	-
Phenols							
1	Cadalin	483-78-3	1488	1636	1.49 ± 1.47	2.60 ± 1.60	-
2	(-)-γ-Cadinene	39029-41-9	1394	1480	2.65 ± 0.62 ^a^	5.70 ± 0.44 ^b^	Woody
3	(1R,4aS,8aR)-1-Isopropyl-4,7-dimethyl-1,2,4a,5,6,8a-hexahydronaphthalene	20085-19-2	1374	1433	1.52 ± 0.78	1.40 ± 0.88	-
4	1-Isopropyl-4,7-dimethyl-1,2,3,5,6,8a-hexahydronaphthalene	16729-01-4	1400	-	31.20 ± 1.24 ^a^	15.63 ± 1.52 ^b^	-
5	Naphthalene,1,2,3,4,4a,7-hexahydro-1,6-dimethyl-4-(1-methylethyl)-	16728-99-7	1463	1515	ND	2.53 ± 0.58	-
6	4-Isopropyl-6-methyl-1-methylene-1,2,3,4-tetrahydronaphthalene	637-69-4	1112	1152	ND	3.54 ± 0.11	Sweet
Ketones							
1	8-Isopropyl-1,5-dimethyltricyclo[4.4.0.02,7]dec-4-en-3-one	1209-91-2	1489	1687	1.08 ± 0.71	1.01 ± 0.5	-
Others							
1	10,11-Epoxycalamenene	143785-42-6	1380	1491	0.53 ± 0.31 ^a^	1.22 ± 0.76 ^b^	-
2	2-[5-(4-Chlorophenyl)-1H-1,2,4-triazol-3-yl]pyrazine	1000387-00-3	1683	-	ND	0.61 ± 0.07	-

CEOP: Cinnamon bark essential oil, CEOY: Cinnamon leaf essential oil. The RI calculated in this study was obtained using an HP-5MS column, the RI obtained from the literature was obtained using an HP-5MS column, the aroma description was obtained from https://www.thegoodscentscompany.com/ (accessed on 5 May 2025). The data are shown as means ± SD; ND, not detected; -, not checked. ^a,b^ Means in the same row with different superscript letters differ significantly (*p* < 0.05).

**Table 3:** Summary of the binding energy and the interaction forces between olfactory receptors and ligands.

Receptors	Ligand	Hydrophobic Interactions	Hydrogen Bonds	Binding Energy (kcal/mol)
OR1A1	Cinnamaldehyde	Asn84, Glu24, Phe177, Val17, Gly16, Lys90, Met81, His85, Leu14	-	−4.39
	α-Caryophyllene	Ala64, Phe61, Ile49, Asn57, Pro138, His56, Leu55, Pro58	-	−5.56
	Borneol	Ile181, His159, Tyr258, Phe206, Val203, Ile105, Met199, Asn155	Gly202, Asn109	−5.01
	Benzaldehyde	Gly16, Met81, Val17, Leu14, His85, Glu24, Phe177	Asn84	−4.24
	Nerolidol	Ile37, Ile40, Pro36, Gly41, Leu299, Ser289, Leu290, Phe286	Pro285	−3.80
	α-Pinene	Ile181, His159, Ile105, Asn109, Val203, Tyr258, Asn155, Gly202	-	−5.38
	2-Hydroxy Benzaldehyde	Asp180, Met104, Ile105, Gly108, Tyr258	Tyr276, Tyr178	−3.77
OR1A2	Cinnamaldehyde	Asn24, Phe27, Val17, Lys90, Phe177, Leu14, Gly16, Val81, Phe31, His85, Phe28	Asn84	−4.28
	α-Caryophyllene	Ile37, Thr40, Leu44, Phe286, Leu290, Leu299, Ser289, Pro285	-	−5.28
	Borneol	Tyr258, Ile105, Val203, Lys109, Gly202, Ile181, Met199	-	−5.04
	Benzaldehyde	Gly16, Phe177, Leu14, Asn84, Val81, Asn24	Val17	−4.06
	Nerolidol	Arg29, Asn292, Ile287, Tyr288, Cys238	Thr239, Ser242, Arg122	−3.78
	α-Pinene	Gly202, Lys109, Ala108, Ile105, Val203, Tyr258	-	−5.47
	2-Hydroxy Benzaldehyde	Phe206, Tyr276, Thr254, Ala108, Ile105, Tyr258	Tyr178	−3.53
OR2W1	Cinnamaldehyde	Ser109, Gly108, Tyr104, Phe73, Tyr278, Phe251, Ile255, Tyr259, Met105	-	−3.98
	α-Caryophyllene	Phe200, Glu196, Tyr259, Pro192, Asn264, Met258, Gly263, Met197	-	−5.93
	Borneol	Leu181, Val199, Met258, Pro182, Tyr259, Asn264	Glu196	−4.92
	Benzaldehyde	Ile255, Met105, Gly108, Phe251, Tyr104, Ser109, Phe73	-	−3.98
	Nerolidol	Glu196, Phe200, Met197, Gln261, Pro262, Tyr259, Pro182, Met258, Val185, Asn264, Leu181	Gly263	−4.41
	α-Pinene	Cys112, Ile206, Tyr259, Val207, Leu159, Met105, Ser109, Tyr104, Ile255	-	−5.25
	2-Hydroxy Benzaldehyde	Phe73, Gly108, Cys108, Cys112, Ser109, Met105, Ile235	Tyr104	−3.81
OR1D2	Cinnamaldehyde	Gly203, Leu199, Leu255, Phe207, Tyr259	Tyr182	−3.49
	α-Caryophyllene	Phe207, Leu199, Tyr182, Ile200, Tyr259, His196, Tyr252	-	−6.29
	Borneol	Tyr259, Leu260, Tyr252, Leu255, Phe207	Cys256	−4.84
	Benzaldehyde	Gly203, Cys204, Phe207, Tyr259, Leu199	Tyr182	−3.54
	Nerolidol	Leu260, Tyr155, Gly203, Phe207, Leu255, Tyr252, Tyr259, Leu208, Cys256	-	−4.49
	α-Pinene	Phe207, Tyr252, Tyr259, Cys256, Leu255	-	−5.14
	2-Hydroxy Benzaldehyde	Cys204, Tyr259, Gly203, Phe207, Leu199, Ile200	Tyr182	−3.26
OR5M3	Cinnamaldehyde	Tyr257, Ala201, Gly202, Leu253, Tyr250, Ile254, Phe205	-	−3.58
	α-Caryophyllene	Ser265, Pro261, Met256, Arg259, Tyr257, Val266, Asn173, Pro181	-	−5.86
	Borneol	Lys270, Gly269, Met256, Pro181, Pro180, His174	Val266	−4.36
	Benzaldehyde	His107, Val106, Phe102, Ala201	Tyr257	−3.54
	Nerolidol	Tyr257, Pro261, Pro181, Val266, Ser265, Gly269	Arg259, Met256	−4.26
	α-Pinene	Phe205, Tyr250, Gly202, Thr206, Tyr257, Leu253, Ile254	-	−4.54
	2-Hydroxy Benzaldehyde	Phe102, His107, Met197, Tyr257, Asp179	-	−3.23
OR5AC2	Cinnamaldehyde	Thr242, Met61, Leu65, Tyr292, Arg295, Ile291, Ser241, Ala245	Asn296	−3.48
	α-Caryophyllene	Leu257, Tyr254, Gln209, Ile258, Phe202, Tyr261, Gly205	-	−6.98
	Borneol	His157, Gly205, Ile208, Ala110, Gln209	Thr111, Ala107	−4.51
	Benzaldehyde	Leu257, Gln209, Tyr254, Ile258, Tyr261	-	−3.51
	Nerolidol	Ile201, Gly205, Val262, Tyr261, Leu257, Cys114, Aln110, Gln209, Thr111	Phe202	−4.17
	α-Pinene	Gly43, Leu50, Ser293, Leu294, Met300, Val299, Leu303, Gly46, Leu47, Pro289	-	−4.69
	2-Hydroxy Benzaldehyde	Ile258, Gly205, Tyr261, Leu257	Gln209	−3.26
TRPA1	Cinnamaldehyde	Gln691, Thr684, Asn687, Phe612, Lys620, Thr624, Ala688	Cys621	−4.13
	α-Caryophyllene	Leu609, Lys610, His614, Phe612, Cys665, Cys621, Ile623	-	−4.91
	Borneol	Lys620, Phe612, Cys621, Ser613	His614, Ser616	−4.32
	Benzaldehyde	Cys621, Cys665, Tyr662, Ile623, Lys610, Leu609, Phe612	-	−3.86
	Nerolidol	Phe612, Lys620, Thr684, Ala688, Glu628, Thr624, Cys621, His614	Asn692, Glu691	−5.16
	α-Pinene	Lys610, Phe612, Leu609	-	−4.32
	2-Hydroxy Benzaldehyde	Leu609, Lys661, Cys621, Phe612, Lys610	-	−3.89
TRPV1	Cinnamaldehyde	Tyr511, Leu515, Leu553, Ser512, Ala566, Tyr554, Gln700, Gly563, Val567, Glu570	Thr556, Arg557	−6.17
	α-Caryophyllene	Ser510, Phe496, Ile493, Phe517, Glu513, Gly492, Ile514, Tyr495	-	−7.51
	Borneol	Ile514, Phe496, Leu506, Phe517	Gly492	−5.82
	Benzaldehyde	Tyr444, Phe488, Tyr441, Val440, Asn437, Tyr487	Tyr555	−5.23
	Nerolidol	Phe496, Leu503, Gly492, Phe517, Tyr495, Ile514	-	−5.94
	α-Pinene	Glu513, Phe517, Phe496, Leu506, Ile514, Gly492	-	−6.23
	2-Hydroxy Benzaldehyde	Tyr495, Glu513, Ile514, Gly492, Phe496, Pro501, Leu506	Ser510	−5.37

-, not detected.

## Data Availability

The original contributions presented in the study are included in the article and Supplementary Materials, further inquiries can be directed to the corresponding authors.

## Supplementary Materials

The following supporting information can be downloaded at: https://www.mdpi.com/article/10.3390/foods14203570/s1, Table S1: *p*-value and VIP-value in essential oils by different parts of cinnamon.; Table S2: Values of cinnamon essential oils sensed by different sensors in an electronic nose; Figure S1: The method for essential oil extraction; Figure S2: Simulation results for molecular docking between cinnamaldehyde and (A) OR1A1; (B) OR1A2; (C) OR2W1; (D) OR1D2; (E) OR5M3; (F) OR5AC2.

## Abbreviations

GC-MS	Gas chromatography–mass spectrometer
VOCs	Key volatile organic compounds
CEOP	Essential oil from cinnamon bark
CEOY	Essential oil from cinnamon leaf
OAV	The odor activity value
PCA	Principal component analysis
OPLS-DA	Orthogonal partial least-squares discrimination analysis
QDA	Quantitative descriptive analysis
ORs	Olfactory receptors
TRPA1	Transient receptor potential A1
TRPV1	Transient receptor potential vanilloid 1
RMSD	Root mean square deviation
RMSF	Root mean square fluctuation
HB	Hydrogen bonds
Rg	Radiuses of gyration
SASA	Solvent accessible surface area
