# Supplementary material for: Compounds of Essential Oils from Different Parts of Cinnamomum cassia and the Perception Mechanism of Their Characteristic Flavors
Source: Foods. 2025 Oct 20;14(20):3570. doi: 10.3390/foods14203570 (PMC12563876; doi:10.3390/foods14203570)
Supplement: Supplementary file 1 [file foods-14-03570-s001.zip › Supplementary material.pdf]

Table S1. P-value and VIP-value in essential oils by different parts of cinnamon.

No	Compound	CAS	RI		CEO		p	VIP
			Calculated value	Literature value	CEOP	CEOY		
Alcohols								
1	τ-Muurolol	19912-62-0	1471	1606	9.83±3.26	4.49±0.95	0.000	1.055
2	Caryophyllenyl alcohol	913176-41-7	1428	-	1.19±1.13	3.59±1.07	0.005	0.992
3	(3E,7E)-1,5,5,8-Tetramethylcycloundeca-3,7-dienol	28446-26-6	1447	1618	1.79±0.95	ND	0.000	1.045
4	Junenol	472-07-1	1457	1588	0.51±0.41	ND	0.001	1.034
5	2,4-Quinolinediol	86-95-3	1482	-	0.45±0.26	ND	0.007	1.002
6	Bicyclo[2.2.1]heptan-2-ol,1,7,7-trimethyl-,(1S-endo)-	464-45-9	1140	1124	0.31±0.19	5.68±0.12	0.613	0.677
7	Ethylene glycol	14912-44-8	1304	1322	0.60±0.30	1.66±0.11	0.056	0.897
8	α-Cadinol	481-34-5	1477	1610	1.45±0.70	6.08±0.70	0.434	0.715
9	Borneol	507-70-0	1140	1136	0.73±0.15	2.48±0.20	0.054	0.850
10	Nerolidol	7212-44-4	1425	1514	ND	10.70±0.80	0.000	1.057
11	α-Bisabolol	30159-13-8	1304	-	ND	4.93±1.47	0.003	1.015
12	(5S,6R,7S,10R)-7-Isopropyl-2,10-dimethylspiro[4.5]dec-1-en-6-ol	72203-99-7	1438	1572	3.27±2.15	ND	0.001	1.042
13	(1S,3aS,4S,5S,7aR,8R)-5-Isopropyl-1,7a-dimethyloctahydro-1H-1,4-methanoinden-8-ol	21966-93-8	1449	-	3.26±2.06	ND	0.001	1.043
Aldehydes								
1	Cinnamaldehyde	104-55-2	1249	1249	701.34±73.67	636.83±34.14	0.000	1.049
2	Benzenepropanal	104-53-0	1150	1160	1.21±1.21	2.78±1.93	0.557	0.339
3	Benzaldehyde	100-52-7	-	-	0.68±0.73	ND	0.004	1.015
4	(Z)-2-Methoxycinnamaldehyde	76760-43-5	1421	1463	2.45±0.35	8.70±1.48	0.001	1.027
5	2-Hydroxy Benzaldehyde	90-02-8	-	-	ND	0.91±0.07	0.000	1.057
Terpenes								
1	Copaene	3856-25-5	1473	1472	45.54±20.82	36.94±1.61	0.000	1.053
2	Cubenene	29837-12-5	1405	1512	7.32±3.06	ND	0.000	1.053
3	α-Calacorene	21391-99-1	1410	1513	6.34±2.62	6.19±1.17	0.001	1.038
4	α-Caryophyllene	6753-98-6	1357	1418	12.46±1.13	9.38±1.91	0.118	1.022
5	γ-Curcumene	451-55-8	1494	1487	0.93±0.36	7.48±0.45	0.001	1.031
6	β-Bisabolene	495-61-4	1391	1485	0.78±0.40	8.06±1.19	0.001	1.030
8	α-Muurolene	10208-80-7	1374	1471	55.82±10.37	26.41±2.34	0.000	1.053
9	γ-Muurolene	30021-74-0	1444	1444	7.63±1.22	ND	0.000	1.056
10	Isoledene	95910-36-4	1280	1373	2.92±0.54	13.60±0.25	0.000	1.046
13	α-Pinene	7785-70-8	-	-	0.46±0.44	1.44±0.90	0.007	0.997
14	Camphene	79-92-5	-	-	ND	4.85±0.11	0.000	1.055
15	1,13-Tetradecadiene	21964-49-8	1454	1385	ND	3.48±1.57	0.002	1.022
16	(E,Z)-α-Farnesene	1000293-03-2	1486	-	ND	1.79±0.89	0.003	1.017
17	(Z)-1-Methyl-4-(6-methylhept-5-en-2-ylidene)cyclohex-1-ene	13062-00-5	1405	1478	ND	6.26±0.49	0.000	1.056
18	Bicyclo[7.2.0]undec-4-ene,4,11,11-trimethyl-8-methylene-,[1R-(1R*,4Z,9S*)]-	118-65-0	1335	1383	ND	5.12±0.12	0.005	1.003
19	Bicyclo[3.1.1]hept-2-ene,2,6-dimethyl-6-(4-methyl-3-pentenyl)-	17699-05-7	1347	1403	ND	5.34±0.42	0.000	1.056
20	Caryophyllene oxide	1139-30-6	1436	1537	ND	8.32±1.92	0.000	1.049

21	Zonarene	41929-05-9	1386	-	ND	8.26±1.26	0.000	1.052
22	(+)-Sativene	3650-28-0	1316	1396	4.29±1.35	2.37±0.38	0.000	1.049
23	(+)Cuparene	16982-00-6	1474	1488	ND	5.26±0.88	0.010	0.984
24	β-Cadinene	523-47-7	1454	1472	3.88±0.21	8.04±0.28	0.009	0.980
25	α-Corocalene	20129-39-9	1459	1605	2.03±1.09	ND	0.000	0.186
26	β-selinene	17066-67-0	1377	1436	0.51±0.24	ND	0.000	1.044
27	(+)-d-Cadinene	483-76-1	1400	1497	64.46±18.02	34.31±0.56	0.000	1.057
28	α-curcumene	644-30-4	1376	1453	0.83±0.19	7.07±2.19	0.001	1.029
29	1H-Cyclopropa[a]naphthalene,1a,2,3,5,6,7,7a,7b-octahydro-1,1,7,7a-tetramethyl-,(1aR,7R,7aR,7bS)-	17334-55-3	1342	1407	ND	6.78±0.23	0.000	1.056
30	1,2,4-Metheno-1H-indene	22469-52-9	1379	1377	4.80±1.34	1.11±0.66	0.000	1.048
Alkanes								
1	Pentane	107-83-5	-	-	3.94±1.29	6.99±0.72	0.028	0.914
2	Cyclohexane	499-97-8	-	-	1.07±0.95	ND	0.002	1.026
3	Cyclopentane	96-37-7	-	-	3.10±2.85	16.79±1.77	0.000	1.049
4	Gossonorol	92691-77-5	1468	1625	ND	7.35±0.65	0.003	1.018
Esters								
1	(E)-Dodec-2-en-1-yl propyl carbonate	1000372-79-9	1453	-	ND	7.41±0.28	0.000	1.056
Aromatic Hydrocarbons								
1	O-Eugenol	579-60-2	1304	1412	0.32±0.06	2.34±0.17	0.000	1.054
2	Phenanthrene,7-ethenyl-1,2	1686-67-5	1626	1884	ND	1.16±0.20	0.000	1.051
Phenols								
1	Cadalin	483-78-3	1488	1636	1.49±1.47	2.60±1.60	0.679	0.262
2	(-)-γ-Cadinene	39029-41-9	1394	1480	2.65±0.62	5.70±0.44	0.073	0.862
3	(1R,4aS,8aR)-1-Isopropyl-4,7-dimethyl-1,2,4a,5,6,8a-hexahydronaphthalene	20085-19-2	1374	1433	1.52±0.78	1.40±0.88	0.013	0.948
4	1-Isopropyl-4,7-dimethyl-1,2,3,5,6,8a-hexahydronaphthalene	16729-01-4	1400	-	31.20±1.24	15.63±1.52	0.000	1.057
5	Naphthalene,1,2,3,4,4a,7-hexahydro-1,6-dimethyl-4-(1-methylethyl)-	16728-99-7	1463	1515	ND	2.53±0.58	0.000	1.048
6	4-Isopropyl-6-methyl-1-methylene-1,2,3,4-tetrahydronaphthalene	637-69-4	1112	1152	ND	3.54±0.11	0.000	1.056
Ketones								
1	8-Isopropyl-1,5-dimethyltricyclo[4.4.0.02,7]dec-4-en-3-one	1209-91-2	1489	1687	1.08±0.71	1.01±0.5	0.000	0.910
Others								
1	10,11-Epoxycalamenene	143785-42-6	1380	1491	0.53±0.31	1.22±0.76	0.521	0.366
2	2-[5-(4-Chlorophenyl)-1H-1,2,4-triazol-3-yl]pyrazine	1000387-00-3	1683	-	ND	0.61±0.07	0.000	1.054

CEOP: Cinnamon bark essential oil, CEOY: Cinnamon leaf essential oil. P values and VIP values from SIMCA. The data was shown as means ± SD; ND, not detected;-,not checked.

Table S2. Values of cinnamon essential oils sensed by different sensors in an electronic nose.

Sensor	Main gas targets	Esponse value		P	VIP
		CEOP	CEOY		
W5S	Nitrogen oxides	20.24±0.07	53.77±0.15	<0.01	2.58
W1W	Inorganic sulfur compounds;many terpenes and sulfur-containing organic compounds	18.61±0.08	28.88±0.04	<0.01	1.43
W2W	Aromatic compounds;sulfur organic compounds	15.18±0.07	18.15±0.01	<0.01	0.77
W1S	A broad range of organic compounds	3.37±0.01	4.98±0.01	<0.01	0.57
W1C	Aromatic organic compounds	0.59±0.01	1.72±0.01	<0.01	0.47
W2S	Aromatic compounds	1.37±0.01	1.94±0.01	<0.01	0.34
W5C	Alkane,aromatic compounds;less polar organic compounds	0.84±0.07	0.74±0.15	<0.01	0.14
W3C	Ammonia,sensor for aromatic compounds	0.87±0.01	0.78±0.01	<0.01	0.14
W6S	Detects mainly hydrogen gas,selective (breath gases)	1.01±0.01	1.05±0.01	<0.01	0.09
W3S	Reacts to high concentrations of methane	1.01±0.01	1.02±0.01	<0.01	0.04

CEOP: Cinnamon bark essential oil, CEOY: Cinnamon leaf essential oil. P value and VIP value from SIMCA. The data was shown as means ± SD.

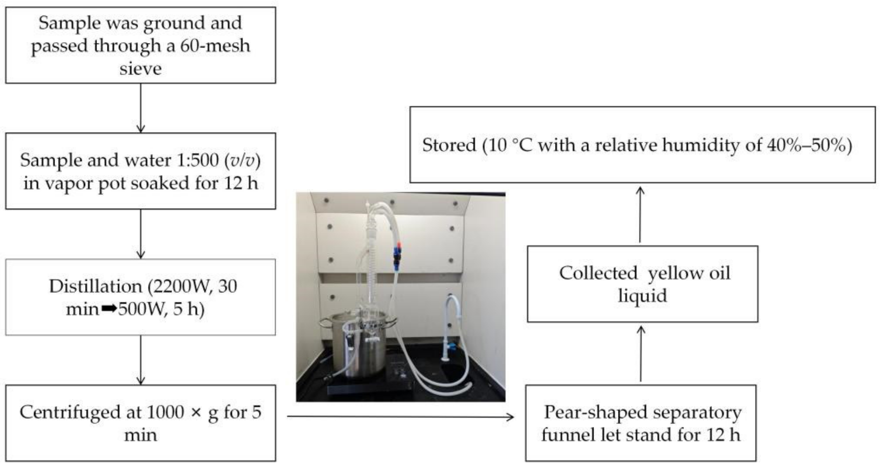

Figure S1. The method for essential oil extraction

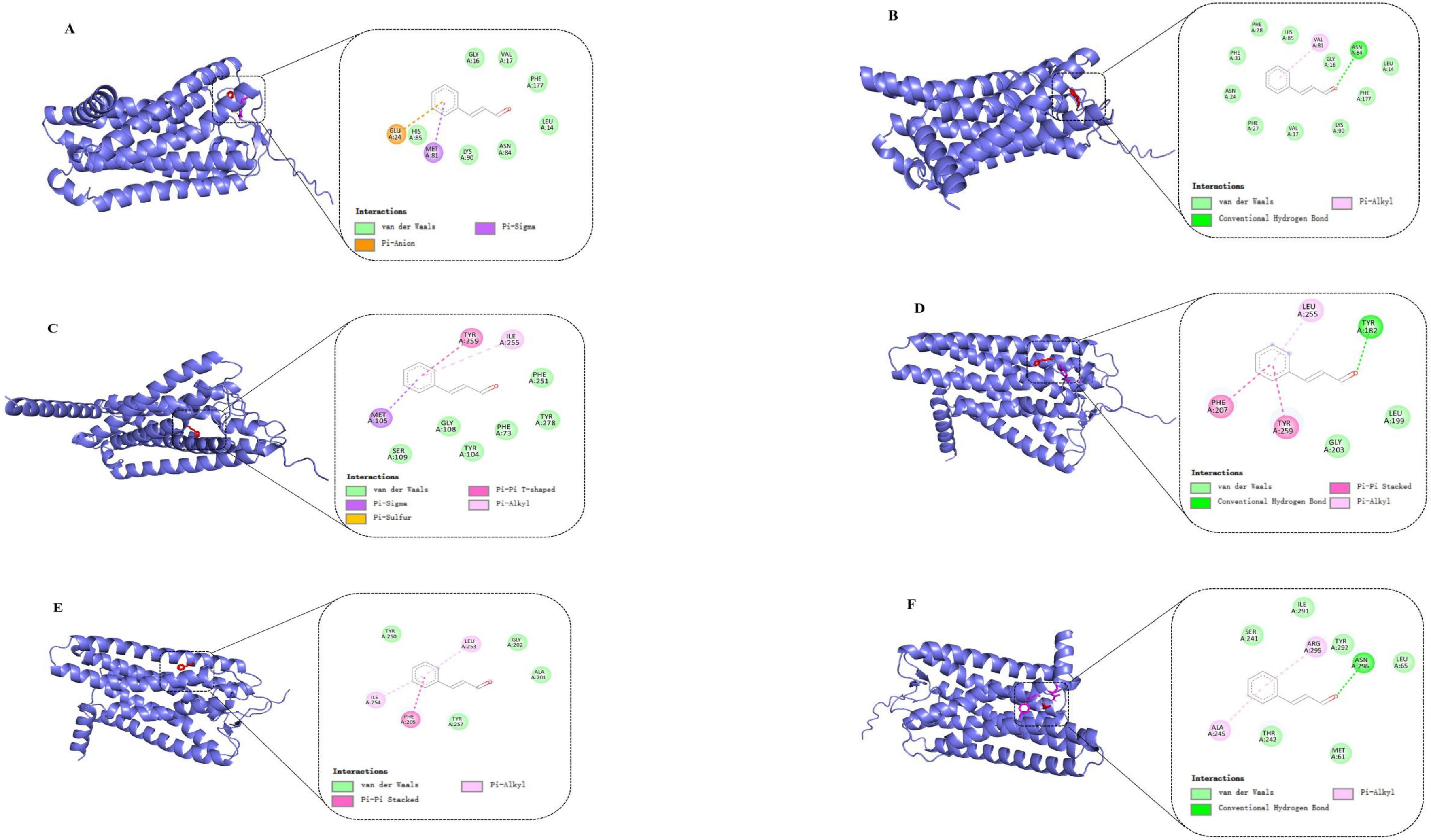

Figure. S2. Simulation results for molecular docking between cinnamaldehyde and (A) OR1A1; (B) OR1A2; (C) OR2W1; (D) OR1D2; (E) OR5M3; (F) OR5AC2.
